# Kubernetes Cluster for Automating Software Production Environment

**DOI:** 10.3390/s21051910

**Published:** 2021-03-09

**Authors:** Aneta Poniszewska-Marańda, Ewa Czechowska

**Affiliations:** Institute of Information Technology, Lodz University of Technology, 90-924 Lodz, Poland; Ewa@kudulab.io

**Keywords:** software production environment, production environment, Kubernetes, Amazon Web Services (AWS), Amazon Elastic Kubernetes Service (EKS), operations automation

## Abstract

Microservices, Continuous Integration and Delivery, Docker, DevOps, Infrastructure as Code—these are the current trends and buzzwords in the technological world of 2020. A popular tool which can facilitate the deployment and maintenance of microservices is Kubernetes. Kubernetes is a platform for running containerized applications, for example microservices. There are two main questions which answer was important for us: how to deploy Kubernetes itself and how to ensure that the deployment fulfils the needs of a production environment. Our research concentrates on the analysis and evaluation of Kubernetes cluster as the software production environment. However, firstly it is necessary to determine and evaluate the requirements of production environment. The paper presents the determination and analysis of such requirements and their evaluation in the case of Kubernetes cluster. Next, the paper compares two methods of deploying a Kubernetes cluster: kops and eksctl. Both of the methods concern the AWS cloud, which was chosen mainly because of its wide popularity and the range of provided services. Besides the two chosen methods of deployment, there are many more, including the DIY method and deploying on-premises.

## 1. Introduction

Microservices, Continuous Integration and Delivery, Docker, DevOps, and Infrastructure as Code—these are the current trends and buzzwords in the technological world of 2020. A popular tool which can facilitate the deployment and maintenance of microservices is Kubernetes. It is a platform for running containerized applications, for example microservices. Kubernetes provides mechanisms for maintaining, deploying, healing and scaling containerized microservices. Thanks to that, Kubernetes hides the complexity of microservices orchestration. It is much easier to satisfy non-functional requirements of microservices deployment by using Kubernetes. An example of such requirements may be: availability, healing, redundancy or autoscaling [1,2,3]. There are two main questions which answer was important for us: how to deploy Kubernetes itself and how to ensure that the deployment fulfills the needs of a production environment.

There are plenty methods of Kubernetes cluster deployment. The methods differ in relation to: how much customization they offer, which clouds they support, how much they cost. Some of the methods has existed since the Kubernetes was created, the other ones, such as AWS EKS, were invented later. There already exist comparisons between different deployment methods of a Kubernetes cluster. However, to the best of our knowledge, these comparisons either: are based on theoretical information (e.g., technical documentation) or are created through a informal, non-academic process and presented as a blog post or they consider only a vanilla cluster.

There exist sources which compare several methods of Kubernetes cluster deployment. However, they are either informal sources (e.g., blog posts or Internet tutorials) or they do not compare the two methods selected in our research or they do not consider the production environment. Therefore, this paper discussing the comparison of two chosen methods offers the aspects not presented before.

Our goal was to apply a practical approach (by really deploying a Kubernetes cluster, not just reading how to do it), to do it in an automated manner (so that the experiment can be easily reproduced) and to ensure that the cluster is ready to be used in a production environment. Thus, the paper aims to compare two methods of deploying a Kubernetes cluster: kops and eksctl. Both of the methods concern the AWS cloud, which was chosen mainly because of its wide popularity and the range of provided services. Besides the two chosen methods of deployment, there are many more, including the DIY method and deploying on-premises.

Any application may comprise several components, for example: a backend server, a frontend server, database. It is common knowledge that deploying an application to a production environment, should obey a set of guidelines. It should be easy to view all the log messages generated by each of the components of the application, the application should be reachable for its end users and also, it would be useful if in a case of any component failure—that component should be available despite the failure. Kubernetes facilitates satisfying such requirements. However our aim is to ensure such requirements for Kubernetes itself. A set of requirements were selected. Then, several ideas were provided on how to meet each of the chosen requirements. This makes us believe that the way in which we deployed the Kubernetes clusters was a formal, documented way.

The process of deploying multiple containers of one application can be optimized through automation. This kind of automation is referred to as orchestration [4]. Kubernetes was built to make deployments easy, to reduce the time and effort which needs to be poured into them. To achieve this goals, Kubernetes offers a wide variety of features [3,5,6,7]:

- creating, destroying, replicating containers,
- rolling updates of containers,
- built-in health checks (liveness and readiness probes),
- autoscaling,
- redundancy and failover—to make the applications deployed on top of Kubernetes more resilient and reliable,
- being provider-agnostic—Kubernetes can be deployed on-premises and in the cloud and in both cases it will provide the same set of features, thus, it may be said that Kubernetes unifies the underlying infrastructure,
- utilizing provider-specific (sometimes referred to as: vendor-specific) features, e.g., AWS load balancer or Google Cloud load balancer,
- self-healing,
- service discovery,
- load balancing,
- storage orchestration.

Our research concentrates on the analysis and evaluation of Kubernetes cluster as the software production environment, especially the production deployment that means such a deployment which targets the production environment. In the framework of this research we made the comparison of two methods of deploying a Kubernetes cluster: kops and eksctl.

Firstly, the paper attempts to stipulate the requirements of a production environment. Then, the requirements are used as comparison criteria to help assess the two methods of deployment. It was expected that one method could be easier to use than the other but also a method could be insufficient to satisfy all the production environment requirements. Furthermore, other criteria were used, such as: cost of both methods and amount of problems encountered. The paper presents the determination and analysis of such requirements and their evaluation in the case of Kubernetes cluster. They are necessary to use as comparison criteria to help assess the two methods of deployment the cluster.

The first novelty of this manuscript is that, while working on it, Kubernetes clusters were indeed deployed on a chosen cloud (AWS) and it was done so in a formal, documented way. First, the 9 requirements of a cluster were gathered, then each of the requirements was planned and then, the deployments were implemented, the clusters were tested and then destroyed. Thanks to deploying the clusters by the authors, it was possible to be sure of exactly which and how many of AWS resources (such as EC2, ALB, EBS) were created by a deployment tool. Based on this information the Section 5.6. was written—it was possible to compare how much a Kubernetes cluster deployed with AWS EKS and kops would cost to run for one month.

The second novelty is that the deployed Kubernetes clusters were not vanilla clusters. The authors undertook the challenge of providing a specification of a production environment. The 9 production environment requirements were collected and an attempt was made to satisfy each of them. Thanks to that such a Kubernetes cluster deployment is much closer to a real-life scenario and represents a more practical approach.

The main contribution of this manuscript is the comparison of two methods of deploying a Kubernetes cluster: kops and eksctl, described in Section 4 and Section 5. They contain the helpful information for anyone committed to a Kubernetes cluster deployment planning. Examples of such information are: time needed to create a production-grade cluster, information on how easy it is to configure a cluster, cost of running a cluster for one month.

The presented paper is composed as follows: Section 2 presents the production deployment requirements—their idea, their classification and description of each type chosen for the further research. Section 3 describes the Kubernetes cluster production deployment requirement and methods. Section 4 deals with available Kubernetes cluster deployment methods: AWS EKS Managed Service using *eksctl* and AWS using *kops* while Section 5 presents the comparison results of the used methods of Kubernetes cluster deployment.

## 2. Production Deployment Requirements—Background and Related Works

A Kubernetes cluster may be defined as a collection of storage and networking resources which are used by Kubernetes to run various workloads [7,8]. Another definition states that a Kubernetes cluster is a single unit of computers which are connected to work together and which are provisioned with Kubernetes components [9]. A cluster consists of two kinds of instances: masters and nodes. An instance can be a virtual machine or a physical computer [1,9,10,11].

Typically, there are two reasons for which multiple environments are in use: to support a release delivery process and to run multiple production instances of the system. The first reason allows having a particular build of an application (e.g., a git commit or a specified version of code) well tested. Such a build has to go through many different environments, e.g., testing, staging and production. When a build does not pass all the stages in the former environments, it will not be promoted to the production environment [12,13].

The second reason for multiple environments is that they are used in order to ensure fault-tolerance (when one environment fails, the other can take over), scalability (the work can be spread among many clusters), segregation (it may be decided to handle a group of customers using one environment and the other group with the other environment, e.g., for latency purposes) [12]. Well-known examples of running multiple production deployments can be Blue-green deployments or Canary deployments [10].

The authors of [14] discuss the set of capabilities required for container orchestration platform for design principles. It also provides a guide to identifying and implementing the key mechanisms required in a container orchestration platform.

Throughout this work, a production deployment means such a deployment which targets the production environment. A list of requirements for a production deployment, gathered through the literature, is as follows:

- Central Monitoring—this is helpful when troubleshooting a cluster [5,8,15,16,17].
- Central Logging—this is a fundamental requirement for any cluster with number of nodes or pods or containers greater than a couple [8,16,18].
- Audit—to show who was responsible for which action [17].
- High Availability—authors of [8] go even further and state that the cluster should be tested for being reliable and highly available before it is deployed into production [5,8].
- Live cluster upgrades—it is not affordable for large Kubernetes clusters with many users to be offine for maintenance [8].
- Backup, Disaster Recovery—cluster state is represented by deployed containerized applications and workloads, their associated network and disk resources—it is recommended to have a backup plan for this data [5,8,17].
- Security, secrets management, image scanning—security at many levels is needed (node, image, pod and container, etc.) [5,8,16,17].
- Passing tests, a healthy cluster–‘if you don’t test it, assume it doesn’t work’ [5,8].
- Automation and Infrastructure as Code—in production environment a versioned, auditable and repeatable way to manage the infrastructure is needed [8,17].
- Autoscaling—if application deployed on a Kubernetes demand more resources, then a new Kubernetes node should be automatically created and added to the cluster [5].

### 2.1. Monitoring as Production Environment Requirement

Monitoring helps to ensure that a cluster is operational, correctly configured and that there are enough resources deployed. Monitoring is also indispensable for debugging and troubleshooting [8]. Moreover, monitoring system provides the historical data that is needed for planning purposes. The monitoring strategy should cover four areas [13]:

- configuring the infrastructure in such a way that it is possible to collect the data,
- storing the data,
- providing dashboards, so that data is presented in a clear way,
- setting up notifications or alarms to let users know about certain events.

Monitoring provides various metrics, e.g., CPU usage, memory use, I/O per disk, disk space, number of network connections, response time, etc. Thus, it is helpful at many different levels: at hardware, operating system, middleware and application level. There is a wide range of available open source and commercial tools to take care of monitoring: Nagios, OpenNMS, Flapjack, Zenoss, Tivoli from IBM, Operations Manager from HP, Splunk, etc. [13]. Solutions recommended for a Kubernetes cluster are: Heapster combined with InfluxDB as backend and Grafana as frontend and also cAdvisor [8,19].

**Figure 1:**
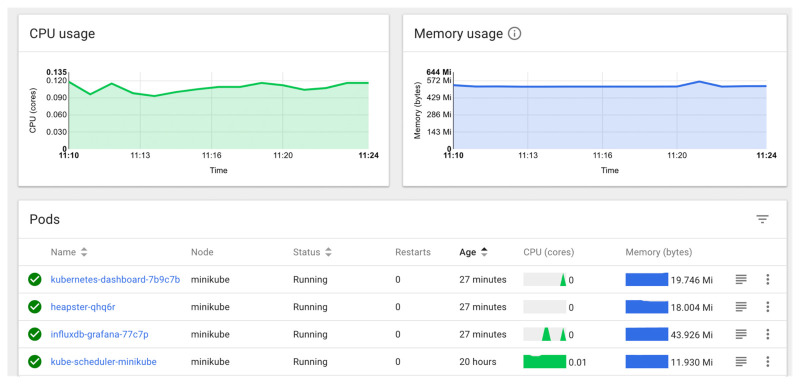
Kubernetes dashboard depicting CPU and Memory usage by Kubernetes pods [20].

Another solution for monitoring is Kubernetes dashboard, which is a built-in solution and doesn’t require any customization. Heapster, InfluxDB and Grafana are great for heavy-duty purposes, whereas Kubernetes dashboard is probably able to satisfy the majority of monitoring needs of a Kubernetes cluster [8,18]. Example dashboard provided by Kubernetes dashboard is presented in Figure 1.

The authors of [21] proposed KRaft, an incorporation of Raft in Kubernetes, a system that manages the containers. The paper presents the evaluation of performance and resource consumption of KRaft together with performance close to Raft executing on physical machines. Moreover, KRaft demands more network transmission then Raft which executed in physical machines needs more processing power and memory.

### 2.2. Logging as Production Environment Requirement

Kubernetes dashboard has also a feature which makes it able to show log messages of a single container deployed on Kubernetes [8]. Centralized logging is essential for a production cluster, because usually there are a lot of pods (and containers) deployed, each generating many log messages. It is impossible to require a Kubernetes administrator to login into each container for the purpose of getting the logs.

The second reason for the importance of centralized logging is that containers are ephemeral—the log messages kept inside the containers would be lost after a container is redeployed. Popular solutions are: Fluentd, Elasticsearch, Kibana [8], Logstash [18], and Graylog [22,23]. It is also important to consider that log messages in Kubernetes cluster are generated from many sources: from end-user applications, nodes, Kubernetes system containers and there are also audit logs in the form of e.g., API server events [24]. For the purposes of auditing, when deploying on AWS, one can use AWS CloudTrail [25].

### 2.3. High Availability as Production Environment Requirement

While administering a Kubernetes cluster, there is a high probability that something will go wrong. For example, components can fail, the network can fail, the configuration can be incorrect, people make mistakes, and software could have bugs. Failure classification was described in [26]. This has to be accepted and a system should be designed in such a way that it is reliable and highly available (HA) despite many problems. Ideas for how to ensure high availability are as follows [8]:

- Redundancy—means having a spare copy of something. Kubernetes uses Replica Sets or Replication Controllers to provide redundancy for applications deployed on Kubernetes. Five redundancy models were summarized in [27]. Some of them require an active replica (running) and other passive (or standby).
- Hot Swapping—can be explained as replacing some failed component on the fly, with minimal or ideally zero down-time. Actually, hot swapping is quite easy to implement for stateless applications. For stateful applications, one has to keep a replica of a component (see redundancy).
- Leader election—it is a pattern used in distributed systems. Whenever there are many servers fulfilling the same purpose to share the load. One of the servers must be elected a leader and then certain operations must go through it. When the leader server experiences a failure, other server can be selected as new leader. This is a combination of redundancy and hot swapping.
- Smart load balancing—used to share and distribute the load.
- Idempotenc—means that one request (or some operation) is handled exactly once.
- Self-healing—means that whenever a failure of one component happens, it is automatically detected and steps are taken (also automatically) to get rid of the failure.
- Deploying in a cloud—a goal is to be able to physically remove or replace a piece of hardware, either because of some issues or because of preventative maintenance or horizontal growth. Often this is too expensive or even impossible to achieve [26]. Traditional deployments on-premises forced administrators to do a capacity planning (to predict the amount of computing resources). Thanks to the on-demand and elastic nature of the clouds, the infrastructure can be closely aligned to the actual demand. It is also easy to scale applications deployed on a cloud, because of the fundamental property of the cloud: elasticity [28].

Furthermore, it may be necessary to test high availability. This can be done by inducing a predictable failure and verifying if the system behaves as expected [8,29]. Such a kind of testing, where one or more cluster nodes or pods is killed is called Chaos Monkey, after the tool developed by Netflix. There are also ready to use tools basing on the idea of Chaos Monkey: chaoskube, kube-monkey, PowerfulSeal [5].

### 2.4. Automation as Production Environment Requirements

When it comes to automation, many guidelines can be found in [13]. The most important guidelines for automation in production environment are as follows:

- Every Change Should Trigger the Feedback Process—means that every change in code should trigger some pipeline and should be tested (including unit tests, functional acceptance tests, non-functional tests). The tests should happen in an environment which is as similar as possible to production. Some tests may run in production environment too [12,13].
- Feedback Must Be Received as Soon as Possible—this also involves another rule: fail fast. This guideline suggests that faster tests (or less resource-intensive tests) should run first. If theses tests fail, the code does not get promoted to the next pipeline stages, which ensures optimal use of resources [13].
- Automate Almost Everything—generally, the build process should be automated to such extent where specific human intervention or decision is needed. However there is no need to automate everything at once [12,13].
- Keep Everything in Version Control—this means that not only application source code but also tests, documentation, database configuration, deployment scripts, etc. should be kept in version control and that it should be possible to identify the relevant version. Furthermore, any person with access to the source code should be able to invoke a single command in order to build and deploy the application to any accessible environment. Apart from that, it should be also clear which version in version control system was deployed into each environment [13].
- If It Hurts, Do It More Frequently, and Bring the Pain Forward—if some part of the application lifecycle is painful, it should be done more often, certainly not left to do at the end of the project [13].
- Idempotency—the tools used for automation should be idempotent, which means that no matter how many times the tool is invoked, the result should stay the same [12].

Presntly, the most essential tools needed to introduce the automated application lifecycle are the following: first, a framework for Configuration Management is needed, e.g., Puppet, CFEngine [12,13], Chef [30], Ansible [31], SaltStack [32]. These tools help to declaratively define what packages should be installed and how should they be configured in a virtual machine or a container or a physical server [13]. They can help prevent configuration drift in a large number of computers. A configuration drift is a difference across systems that were once identical. It can be imposed by a manual amendment and also by automation tools which propagated a change only to some of the instances [12]. There are also stack-oriented tools, which follow the same declarative model: Terraform [33] and CloudFormation [12]. Another type of needed tools is a building server, where the examples are: Jenkins, GoCD, Travis.

### 2.5. Security as Production Environment Requirement

Security is another essential aspect of production deployment and it touches many levels. A node breach is a very serious problem and it can happen by someone logging to the instance or having physical access to it. The latter is easily mitigated by not deploying on bare-metal machines but on a cloud instead [8]. The former demands some hardening done. There are several ideas that can be implemented for a Kubernetes cluster specifically listed below:

- Ensuring that data is encrypted in transit by using secure API server protocol (HTTPS instead of HTTP) [8].
- Ensuring proper user and permissions management by configuring authentication, authorization, security accounts and admission control in API server [8]. When setting up authorization, it is wise to apply the principle of least privilege. This principle recommends that only the needed resources or permissions should be granted [5].
- Using Role-Based Access Control (RBAC) to manage access to a cluster [5,34,35].
- Ensuring security keys management and exchange by implementing for example automated key rotation.
- Ensuring that used Docker images are neither malicious (deliberately causing some harm) nor vulnerable (allowing some attacker to take control) by keeping them up-to-date and maintaining them instead of using the publicly available ones or by using a private Docker registry [8].
- Using minimal Docker images because the fewer programs there are installed in an image, the fewer potential vulnerabilities there are [5].
- Maintaining a log or audit system [8].
- Using network policies which act in a white list fashion and can open certain protocols and ports [8].
- Using secrets. Kubernetes has a resource called: secret, but the problem is that Kubernetes stores secrets as plaintext in etcd. This, in turn, means that steps should be taken in order to limit direct access to etcd [8].
- Preferring managed services, because they will have many security measures already implemented [5].
- Avoid running processes as root user in Docker containers [5].
- Using available programs for security scanning [5].

### 2.6. Disaster Recovery as Production Environment Requirement

Disaster recovery can be understood as the process which an organization has to undergo after a service disruption happened in order to resume normal services. It is vital to know what actions are necessary to overcome the disaster. This set of predefined procedures is known as Disaster Recovery Plan, DRP. Furthermore, disaster recovery is not the same as fault tolerance. The latter ensures that a system will withstand and resist the failure [11].

Disaster recovery is an essential requirement of any business where continuity matters. In order to plan disaster recovery well the following key parameters should be considered: the initial cost, the cost of data transfers and the cost of data storage. Significant costs may be a reason why, in the past, around 40–50% of small businesses had no DRP and did not intend to change this. However, cloud computing provides affordable solutions, because of the employed model “pay-for-what is used”. Another advantage of the cloud is that it is fairly easy to use resources deployed in multiple geographical areas. This is desired, because one the major concepts in a DRP is the geographical separation of the system servers and its backup [36].

Key metrics that can be taken into consideration while planning disaster recovery are [11,36]:

- Recovery Point Objective (RPO),
- Recovery Time Objective (RTO).

Cloud services mitigate some risks that persistent data storage has. For example cloud services provide high-available data storage by replicating it across different geographical locations. However, replication is not the same as backup and it does not protect against accidentally deleting a volume or against a misconfigured application overwriting the data. Thus, backup is still needed. In order to backup Kubernetes, the etcd database has to be backed up. Apart from that, each application deployed on top of Kubernetes should be backed up on its own [5]. There are already available services that help with Kubernetes backup: Velero [5].

### 2.7. Testing as Production Environment Requirement

Before the production Kubernetes cluster is ready for end-users, it must be verified that it works and is healthy. Every component and every node should be tested proving that it is working as expected. Sometimes, applications expose a custom health endpoint [18], e.g., kube-scheduler does that. Thanks to that, it is possible to verify regularly that a service is performing correctly by sending a request to the health endpoint. Usually, a HTTP response code of 200 indicates correct status of the service [37]. Kubernetes can monitor the health endpoints with liveness probes. Based on a specified health endpoint response, Kubernetes can restart the faulty container [38].

This section described the production environment requirements together with the example ideas of how to satisfy most of them. Therefore, it is now possible to choose the requirements for Kubernetes cluster deployment.

## 3. Kubernetes Cluster Production Deployment Requirements and Methods

The practical planning and designing the production deployment consider the following activities: capacity planning, choosing which requirements to satisfy and taking different deployment and infrastructure related decisions.

There are numerous requirements for a production deployment of a Kubernetes cluster. In general the companies, which deploy Kubernetes and similar systems, obey some set of best practices, dedicated to these companies only. Thus, the requirements presented in this work do not exhaust the topic.

In the empirical part of our study, the most important requirements were attempted to be satisfied:

- healthy cluster,
- automated operations,
- central logging,
- central monitoring,
- central audit,
- backup,
- high availability,
- autoscaling,
- security.

The set entails nine requirements. We determined the acceptance criteria needed to satisfy each of the requirements. Based on this, a plan for Kubernetes cluster deployment was created.

### 3.1. Healthy and Usable Cluster

Unless all of the Kubernetes control plane components are healthy, it will need to be fixed. It should not be a problem with a managed Kubernetes services, but for self hosted clusters, this should be checked. To monitor the health of a Kubernetes cluster, the following could be checked: number of nodes, node health status, number of pods per node and overall, resource usage/allocation per node, and overall [5].

To verify that an application can be deployed on top of a Kubernetes cluster, a simple Apache server will be deployed. A Helm Chart will be used [39]. Then, Bats-core tests will be run to check that the Apache server works (that the Apache endpoint is reachable from a remote machine).

In order for a cluster to be healthy and usable, the following acceptance criteria must be met:

- the cluster should be tested with Bats-core (i.e., particular number of worker nodes should be deployed, particular Kubernetes version should be used)),
- the Kubernetes API Server endpoint should be reachable for the end users under a domain name (i.e., an end user can connect with the cluster using kubectl),
- it should be tested that an application can be deployed on top of a Kubernetes cluster (i.e., an Apache server will be deployed and tested).

### 3.2. Automated Operations

To be able to perform automated operations, a Kubernetes cluster configuration and commands needed to manage the cluster should be stored in a version control system. This is the goal of Infrastructure as Code. Such a solution will also allow cluster changes to be applied through reviewable commits and in turn it will allow avoiding possible collisions from simultaneous changes being made.

To automate the operations, a Bash script will be used. It should be possible to use this script in order to: create, test and delete a cluster. It is essential to obey the Infrastructure as Code policy, because it facilitates repeating the operations and the whole experiment of a cluster deployment. Although in this work no CI server is going to be used, it should be possible to invoke the automated commands (by using the Bash script) in a potential CI pipeline.

To satisfy the Automated Operations requirement, the following measures will be applied:

- all the code and configuration needed to deploy a cluster will be stored in a Git repository,
- cluster will be configure with YAML file,
- Bash script will be used to create, test and delete a cluster,
- Helm tool will be used to automate a test application deployment,
- it will be possible to choose between two deployment environments: testing and production (templating mechanism with Bash variables will be used).

### 3.3. Central Logging, Monitoring and Audit

The plan here is to simply use AWS CloudWatch service. It should be easy to enable when using eksctl. It is not enabled by default due to data ingestion and storage costs [40].

To satisfy the Central Monitoring requirement, we plan to use AWS CloudWatch. There also exist a solution—a program called: Kubernetes Metrics Server. In addition, even though it is a server which provides source of container resource metrics, it should not be used as an accurate source of resource usage metrics. Its main usage is for CPU/Memory-based horizontal autoscaling and for automatically adjusting/suggesting resources needed by containers. Thus, it will not be used for Central Monitoring.

This requirement will be simply met by using the AWS CloudTrail service. This service should show us which user is responsible for what changes made to the AWS resources used. Besides, AWS CloudTrail can be also used to detect unusual activity in the AWS accounts.

### 3.4. Backup

To implement and test backup, Velero tool will be used. First, Velero will be used to create a backup. Then a disaster will be simulated: a whole Kubernetes namespace will be deleted with all the resources deployed inside. The last phase is the backup restore phase. It is expected that the namespace and all the resources in it will be restored.

### 3.5. Capacity Planning and High Availability

The needed capacity, meaning: number of machines and IT resources (CPU, Memory) must be discussed. The important thing is that the to-be-deployed Kubernetes cluster, should have the minimal amount of capacity, but still it should be representative for a production environment. Here chicken-counting (0, 1, many) comes to the rescue. Applying the chicken-counting technique means that if a production site has 250 web servers, 2 should be enough to represent it [13]. Since a Kubernetes cluster consists of a master node and worker nodes, it was decided that a representative cluster would be made of two worker nodes. One worker node will be created together with cluster creation and the second worker will be added automatically by the Autoscaler. When it comes to number of master nodes needed, one is enough for the minimal deployment. However in order to achieve high availability at least two master nodes are needed. However, having an odd number is recommended for master nodes to avoid issues with consensus/quorum. Thus, at least three master nodes are needed [41,42].

### 3.6. Autoscaling

The idea here is to test whether a cluster, which consists of master nodes and worker nodes, will be able to, by itself, create and delete another worker node. This decision, whether to scale in or out, should be taken according to how many resources there are available for pods. The demand for more resources will be artificially created by increasing the value of replicaCount in the Apache Helm Chart [39].

### 3.7. Security

There are many security measures that could be applied on a cluster. In order to stay secure, access to the Kubernetes cluster should be limited. Any Kubernetes deployment on AWS will have to deal with networking.

Virtual Private Cloud (VPC) is an AWS resource, providing the networking services. When a VPC is created, it means that a virtual private network is created and assigned to a particular AWS account. AWS resources can be launched into that VPC. Each VPC has a CIDR block assigned. The VPC cannot span more than one AWS Region. The VPC can be divided into subnetworks (or subnets). One subnet cannot span more than one Availability Zone. Subnets can be either public or private. If a subnet’s traffic is routed to an internet gateway (which is an AWS resource), the subnet is known as a public subnet. Otherwise, a subnet is private [43]. This means that public subnets have access to the Internet.

By default, both kops and eksctl deploy Kubernetes clusters using public subnets. This means that anyone who knows the URL of the Kubernetes master node, can access its API Server. Additionally, if one has a private SSH key, then they can SSH login into the master node. A simple way to restrict access to the cluster is to use Security Groups. Security Groups rules will be used to limit SSH access to the master and worker nodes and also to limit access to API Server endpoint [44]. In result, only one IP address should be able to access the endpoints.

The deployments operated by a bigger team of many Kubernetes cluster administrators requires that one could use a NAT Gateway or NAT instance IP address as a single allowed IP address. Furthermore, a bastion host could be used to securely SSH login into the master node [45].

To summarize: in order to make the cluster more secure, access to the cluster should be restricted. Only one IP address will be allowed to connect.

## 4. Available Kubernetes Cluster Deployment Methods

Kubernetes is not trivial to deploy. In order to deploy a usable cluster, there are at least two machines needed: one master and one node. On each of the machines, several components must be installed.

Fortunately, Kubernetes is a popular tool and many methods of deploying it are already described in the literature. The available methods may be divided into three categories:

- self-hosted solutions, on-premises,
- deployment in a cloud, but not using Managed Services,
- deployment in a cloud, using Managed Services.

According to the authors of [5], the best solution is to use Managed Services. The authors argue that, thanks to this method, one can get a fully working, secure, highly available, production-grade cluster in a few minutes and for a little price. Managed Services are certainly a good way to try Kubernetes out. Then, if one wants to do something non-standard or to experiment with the cluster, then one could choose a custom or a hybrid solution. The self-hosted, deployed on-premises way is recommended if the following qualities are of a great importance: price, low-network latency, regulatory requirements and total control over hardware [8].

Managed Services offer many features, such as built-in autoscaling, security, high availability, having serverless options. However, they may be more expensive (for example when using AWS EKS, one has to pay $0.10 per hour for each Amazon EKS cluster [46]) and less customizable. Custom solutions allow the Kubernetes administrator to broaden their knowledge and grasp the deep understanding on what is going on under the Kubernetes hood (i.e., one can customize how the Kubernetes control plane is deployed or set up a custom network topology). There is no one right answer which fits all the use cases. It is always advised to do one’s own research and to try and experiment with the existing methods.

Furthermore, different categorization may be applied. For instance, the deployment methods may be categorized by the tools:

- using web interface of a particular cloud, e.g., AWS Management Console (supported by AWS),
- using command-line tools officially supported by a particular cloud, e.g., awscli or eksctl (supported by AWS),
- using command-line tools designed exactly to deploy a Kubernetes cluster, but not limited to one particular cloud, e.g., kops,
- using command-line tools, designed for managing computer infrastructure resources, e.g., Terraform, SaltStack.

However, our focus is on the two particular methods, which were evaluated for the production environment of software deployment:

- deploying on AWS, using AWS Managed Service (AWS EKS), using eksctl which is a AWS supported official tool,
- deploying on AWS, not using any Managed Service, using kops which is a command-line tool, not officially supported by any cloud, but designed exactly to deploy a Kubernetes cluster.

### 4.1. Deployment Method: On AWS EKS Managed Service Using Eksctl

The goal of eksctl method is to have the Kubernetes control plane managed by AWS. This means that it is AWS which is responsible for managing Kubernetes master components, such as: API Server, kube-scheduler, kube-controller-manager, and also Etcd. The control plane should be already highly available, thanks to being deployed across multiple Availability Zones (AZ) [43,45,47].

The control plane being already highly available means that there should be at least two API server nodes and three etcd nodes that run across three Availability Zones within a Region. In addition, AWS EKS is responsible for automatically detecting and replacing unhealthy control plane instances. There exists a Service Level Agreement (SLA) especially concerning AWS EKS. It is a policy which governs the use of the Amazon Elastic Container Service for Kubernetes. The Kubernetes API is exposed via the Amazon EKS endpoint which is associated with the cluster. The cluster control plane is fronted by an Elastic Load Balancing Network Load Balancer and also all the networking is taken care of to provide connectivity from the control plane instances to the worker nodes. Thanks to that, the AWS EKS user can access the Kubernetes API Server [48].

To use AWS EKS, there are two ways supported: using eksctl CLI or using AWS Management Console and AWS CLI. Both of them demand installing AWS CLI. In this work, the method with eksctl CLI is applied. Eksctl is officially the CLI for AWS EKS, endorsed by AWS, though it was launched by WeaveWorks. The cluster can be configured with a YAML file and also by setting eksctl CLI flags.

**Figure 2:**
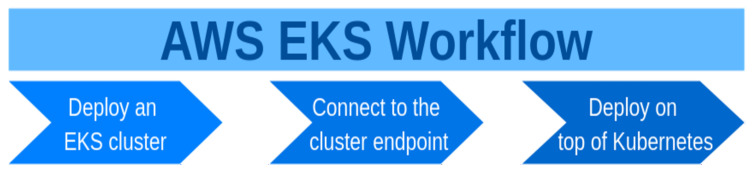
Schema presenting the stages of working with AWS EKS.

After the cluster is created and after a connectivity with a Kubernetes cluster endpoint is established, it is now possible to deploy applications on top of the cluster. The Figure 2 depicts the stages of working with AWS EKS.

### 4.2. Deployment Method: On AWS Using Kops

Kops stands for Kubernetes Operations. It is a command-line tool which allows creating, destroy, upgrade, and maintain production-grade, highly available, Kubernetes clusters. It supports multiple clouds: AWS (officially), GCE and OpenStack (in beta support) and VMware vSphere (in alpha) [49]. Furthermore, it can be used from multiple operating systems: Linux, Mac and Windows [50]. Kops is one of the recommended ways to setup a Kubernetes cluster and it is a tool which can be used to a create a production environment [9].

As with eksctl, kops can also create a Highly Available Kubernetes cluster. However, it demands more work than eksctl. The commands which allow creating a cluster on AWS are:

$ kops create cluster $ {CLUSTER_NAME} -state \

‘‘ s3://$ {K8S_EXP_KOPS_S3_BUCKET}’’ -cloud=aws \

-zones=us-east-1c

$ kops update cluster $ {CLUSTER_NAME} -state \

‘‘ s3://$ {K8S_EXP_KOPS_S3_BUCKET}’’ -yes

Before these commands can be run, one has to provide some minimal DNS configuration via Route53 (an AWS resource responsible for networking), set up a S3 bucket (another AWS resource, responsible for storage) to store the cluster configuration [8] and also configure the AWS IAM user (an AWS resource responsible for access management) and create a SSH key pair [41,44,51]. Amazon S3 is an AWS service which provides object storage. AWS IAM is an abbreviation from AWS Identity and Access Management and it is responsible for managing access to AWS services and resources.

**Figure 3:**
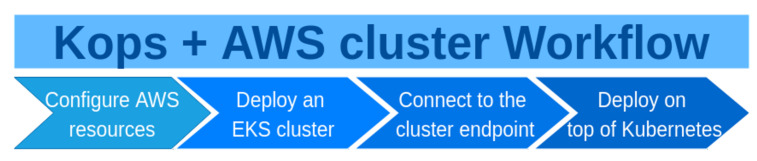
Stages of working with Kubernetes cluster deployed on AWS with kops.

The stages of working with a Kubernetes cluster deployed on AWS with kops are similar to the stages of working with eksctl, but there is the additional first stage (Figure 3).

Kops has been around a long time as an AWS-specific tool, but now it also supports other clouds [5,49]. Its main features such as: automated Kubernetes cluster deployment, highly available master or adding a variety of custom Kubernetes addons [22,52] indicate that kops is an attractive tool, worthy to at least try out.

### 4.3. Troubleshooting Any Kubernetes Cluster

This subsection presents the ideas how to troubleshoot any Kubernetes cluster. The ideas were gathered during the empirical work of our research.

There are some general guidelines which help to debug the cluster. When a cluster is self-hosted and one is fully responsible for master and worker nodes deployment, then one should verify whether any node is still functional. It means that problems, such as a corrupt file system, kernel deadlock, problems with the Docker Daemon, or problems with hardware may occur and should be mitigated. A solution to detect such problems may be: a node problem detector pod [8]. One should also run the following commands to check if all the worker nodes are running and if the cluster is generally reachable:

$ kubectl get nodes

$ kubectl cluster -info

Another level of troubleshooting is debugging the applications deployed on top of the Kubernetes cluster. Some ideas are described on the official Kubernetes documentation website [53,54]. For example, one can get details of a pod with the following commands:

$ kubectl describe pods $ {POD_NAME}

$ kubectl logs $ {POD_NAME}

Even the pod status may provide us with enough information, what steps should be taken next. If a pod is in Pending status, then it could mean that there are not enough resources in the cluster (e.g., the worker nodes use too small EC2 instance types or there are too few worker nodes). A pod may be also in status CrashLoopBackOff which indicates that a container is repeatedly crashing after restarting. There may a problem also that a Docker image for a container cannot be downloaded and a pod may be in state ImagePullBackOff or ErrImagePull [55].

It is also always a good idea to read log messages (so access to log messages should be always supplied). The following Kubernetes components log messages may be helpful [56]:

- logs from kube-apiserver, which is responsible for serving the API,
- logs from kube-scheduler, which is responsible for making scheduling decisions,
- logs from kube-controller-manager, which manages replication controllers,
- logs from kubelet, which is responsible for running containers on the node,
- logs from kube-proxy, which is responsible for service load balancing.

Besides all the troubleshooting ideas presented earlier, one can also visit solutions dedicated entirely to AWS EKS clusters [57] or to eksctl [58]. Still, some problems may be tough to handle. However, even then, there are always measures that could be applied to move further into the correct direction. One may ask a question on Stack Overflow or talk to a Kubernetes team on Slack or write on a Kubernetes forum or file a bug on the Kubernetes Github.com project website [59].

To summarize: there are various ways how to debug a Kubernetes cluster or an application deployed on top of it. Some steps may be applied to any Kubernetes cluster, other concern only the clusters deployed using a particular method.

## 5. Comparison of the Used Methods of Kubernetes Cluster Deployment

Several comparison criteria are used in order to compare the two chosen methods of a Kubernetes cluster deployment. Even though the two methods: using kops and using eksctl, help to deploy a Kubernetes cluster, they have some differences. For example: kops works for many clouds (e.g., AWS, GCP), whereas eksctl supports only AWS. Another difference is the default AMI(Amazon Machine Image). kops chooses Debian Operating System, while eksctl uses Amazon Linux 2. These AMIs are, however, only defaults, one can always choose another AMI.

**Figure 4:**
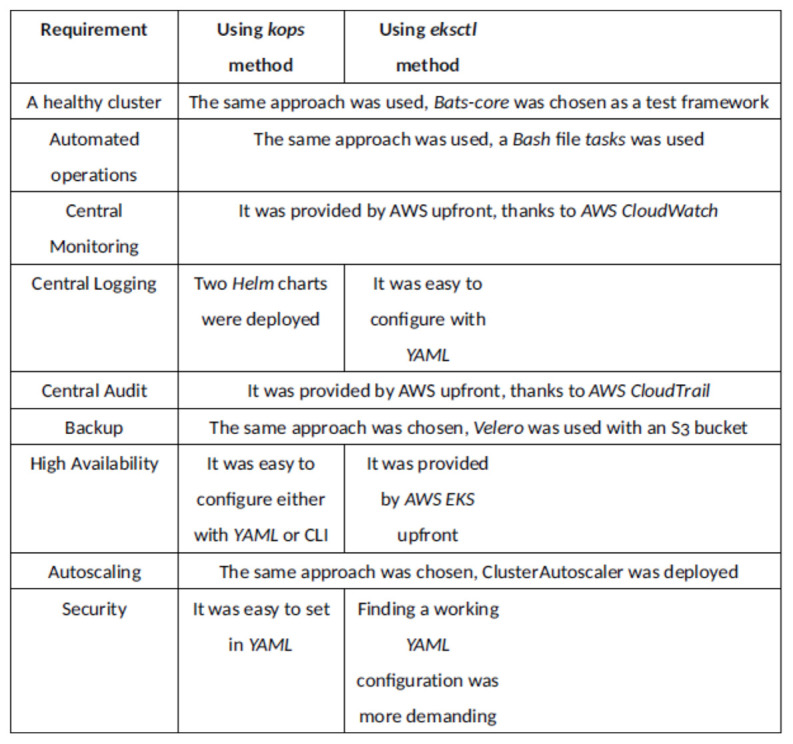
Comparison of how each production requirement was satisfied using kops and eksctl.

There were nine production environment requirements which were supposed to be met by both tested methods. All of them were met. This means that both of the methods can be applied in production use cases. Figure 4 provides a brief explanation how each of the requirements was satisfied for each method. Many requirements were handled in the same way for both methods. Subjectively, the hardest requirement to meet was: security, using eksctl. There was a problem with finding a working YAML configuration and also ensuring that cluster was healthy. Eksctl provides a more automated approach. For example the AWS CloudWatch LogGroup, needed for central logging, was automatically created when using eksctl, but not when using kops. Also, eksctl provides the cluster which is already Highly Available. On the other hand, kops provides more flexibility. The master node can be accessed with SSH when using kops and not when using eksctl. When using kops, it is possible to see all the kube-system namespaced pods, which is not the case with eksctl. When using eksctl no parameters of the control plane components can be set, with kops it is possible.

### 5.1. Time of Chosen Kubernetes Cluster Operations

One of the criterion used to compare the two deployment methods was time. The time of several operations concerning Kubernetes cluster management was measured. Each of the operation was performed several times and the mean value was used. Time was measured separately for a minimal working cluster and for the full cluster. The minimal cluster here means such a cluster that is usable for end user and utilizes the default configuration (central monitoring, central audit, healthy cluster, automated operations are met). The production-grade cluster includes also the following requirements: HA, central logging and security. About 5 min of difference between creating a minimal and full cluster using eksctl was caused by setting the security measures. The full cluster here does not include backup and autoscaling, because meeting these requirements is negligible in the terms of time and takes the same time for kops and eksctl. Testing a cluster involves running the automated tests—verifying that the cluster is healthy. It does not involve testing each of the production deployment requirements. Both the minimal and full clusters use t2.micro for kops and t2.small for eksctl for worker nodes. The time observed for the cluster operations is given in Table 1.

The files’ names used to perform each cluster operation are demonstrated in Table 2. The files can be found in the public Git repository https://github.com/xmik/MastersThesis (accessed on 31 January 2021).

In production deployment using eksctl, there were one command used to generate cluster configuration (eksctl create cluster) and to deploy the cluster. This command did not return (meaning: it waited) for the cluster to be ready. First, the control plane was deployed, then the worker nodes. For each of these two tasks (deploying control plane and deploying worker nodes) a separate CloudFormation stack was used. It was done automatically by AWS EKS. The control plane is entirely managed by AWS and it is run across three AWS availability zones in order to ensure high availability. The end user has even no access to the control plane, meaning: there is no EC2 instance with master node visible and when listing all Kubernetes nodes, only the worker nodes are visible.

A few sources claimed that the creation of Kubernetes cluster on AWS EKS should take 10–15 min [60,61] but in this particular case it took about 20 min (Table 1). Also, to the best of this work’s author knowledge, there was no command which allows reconfiguring the cluster using all settings from the YAML configuration file. This means that when there was a need to change e.g., tags of the AWS resources, the cluster must have been deleted and created from scratch.

To make the cluster more secure, it was decided to restrict the SSH access and kubectl access. Searching through eksctl configuration, there was no setting which allowed to whitelist the IP addresses which are allowed to ssh login to worker nodes. However, blocking all the SSH access was available and it was chosen. This means now that a cluster administrator has only kubectl access to cluster and no ssh access at all (because ssh access to worker nodes is blocked and access to master nodes is not given by design of AWS EKS). It is acknowledged that sometimes, not being able to ssh to the node may make troubleshooting more difficult, but generally system and EKS logs contain enough information for diagnosing problems. The second security requirement was to restrict the access to cluster through kubectl. By default, eksctl exposes the Kubernetes API server publicly but not directly from within the VPC subnets. This means that the public endpoint is by default set to true and the private endpoint to false [62].

There were problems finding a working configuration—the main setting was publicAccessCIDRs. Thus, only one IP address should be allowed to communicate with the cluster through kubectl. It was tested that the command kubectl worked from such an IP. When the same command was run from a different machine, with a different IP, this command reached a timeout. Whenever there is a timeout when reaching an AWS endpoint, it may be caused by a Security Group not allowing such access. Thus, this proved that the kubectl access was restricted and that security measures were working. Creating the EKS cluster with all the security settings took longer—about 25 min (Table 1).

The testing procedure in eksctl was the same as with kops. Different command was invoked (because all the commands to run with eksctl were put into one file), but all the Bats-core tests were the same. Even the same Bats-core files were used. There was obviously no *kops validate cluster* command run, because that command was dedicated to kops clusters only. Running all the tests took slightly less than 7 min. It took long to have a working Load Balancer.

Deleting a cluster command usually took about 13 min. It deleted almost all AWS resources, but not *CloudWatch LogGroup*. Not deleting the *LogGroup* may be a good thing, because one may be interested to study the logs after the cluster is long gone.

To summarize, it is clearly visible that operating a Kubernetes cluster is faster with kops. The time of operations is an important criterion, because firstly, the faster it is, the faster one can get the feedback and experiment with the cluster. In addition, secondly, longer creation and deletion times mean longer time of keeping AWS resources created, which in turn means that they cost more.

### 5.2. Additional Steps Needed to Create a Kubernetes Cluster

To deploy the clusters, many tools were needed. However, these tools were packaged inside a Docker image. Therefore, the development environment, provided by the Docker image was reliable and easily reproducible. It could be destroyed and recreated any time. However, apart from the development tools, there were also some prerequisites needed to be deployed for the production-grade clusters.

When using kops, one has to use and create an S3 bucket. Kops needs this bucket to store cluster configuration. Contrary to that, AWS EKS does not need any additional AWS Resources created beforehands. However, this work attempted to deploy Kubernetes clusters in a production environment. In order to satisfy the backup requirement, Velero tool was used. Velero needs some location to store backups. Therefore an S3 bucket was needed for both deployments.

To summarize, both methods require the same steps to work in a production environment specified in this work, but the S3 bucket was used by choice and it could be replaced with some other storage solution.

### 5.3. Minimal EC2 Instance Type Needed for a Kubernetes Worker Node

Various EC2 instance types cost various amounts of money. Thus, it is preferable to use the cheapest possible instance type. For this reason, the minimal EC2 instance type was needed for a Kubernetes worker node, in a production ready cluster. To meet production environment requirements, this number of pods is needed to be deployed on a worker node (additionally to the pods deployed by default by eksctl and kops):

- three pods for a cluster created with eksctl (one pod for testing, one pod for backup and one pod for autoscaler),
- five pods for a cluster created with kops (one pod for testing, one pod for backup, one pod for autoscaler, two pods for logging).

As far as eksctl method is concerned, there are by default four pods deployed on a worker node. Thus, altogether, there have to be 4 + 3 = 7 pods deployed on a worker node. The EC2 instance types t2.nano and t2.micro allow for maximally four pods on one EC2 instance [63]. The next, bigger instance type is t2.small and it allows for 11 pods. The conclusion is that when it comes to the number of allowed pods on a worker node, the minimal EC2 instance type is t2.small.

Another thing to consider is whether t2.small is enough when it comes to EC2 CPU and memory resources. This was checked empirically, by deploying a cluster with eksctl and then deploying the three pods needed to satisfy the production environment requirements:

eks$ export K8S_EXP_ENVIRONMENT= testing

eks$ ./tasks_create

eks$ ./tasks_enable_velero

eks$ ./tasks_enable_as

eks$ ./tasks_test

All the seven pods were in the status—Running—and the tests were successful. Thus, it may be concluded that when in comes to EC2 instance CPU and memory resources, the EC2 instance type t2.small is enough.

The third aspect is that it is possible to deploy some pods on a master node or mark the master node as schedulable only by Kubernetes system components [38,64]. However, this is not the case for AWS EKS. In AWS EKS, the control plane is locked down and scheduling any workloads on the control plane nodes is forbidden [65]. Contrary to the AWS EKS cluster, there are no hardcoded limits for the number of pods when using kops. From the performed experiments, it was tested that the EC2 instance type t2.micro is too small. The experiment was repeated with the EC2 instance type t2.small for a worker node and with deploying all the services needed for a production environment:

kops$ export K8S_EXP_ENVIRONMENT= testing

kops$ ./tasks_create

kops$ ./tasks_enable_velero

kops$ ./tasks_enable_as

kops$ ./tasks_enable_logging

kops$ ./tasks_test

The tests were successful and all the pods were in status Running. Thus, it may be concluded that the minimal EC2 instance type is t2.small. To summarize, both methods of deploying Kubernetes clusters kops and eksctl allow worker nodes minimally of EC2 instance type: t2.small, in order to satisfy the production deployment requirements. However, for experimental environments, kops allows for smaller types (t2.micro can be used).

### 5.4. Easiness of Configuration

With kops, it was harder to generate the config file. Kops’ documentation recommends that one should use kops create cluster command instead of trying to generate it by hand. Using this command, one needs to create an S3 bucket first, the configuration file is put there and then, the object hosted on an S3 bucket can be exported to a local file. On the contrary, with eksctl, it was easier to generate the YAML file and it was done by hand.

On the other hand, kops allows for configuring the parameters of the control plane components, while eksctl does not. Another thing is that kops allows using Golang templates [66]. Thus, it is possible to use one template file and use it, for example, to deploy into two different environments: testing and production. However, since such templating solution was not possible with eksctl (to the best knowledge of authors), template files with Bash variables were used. It was easier to manage the template files for kops and for eksctl in the same way. This solution worked well and accomplished its purpose.

Considering the encountered problems, there was an unexpected error, when creating the cluster with eksctl and applying the security measure to restrict the IP addresses which can access the API server. It was, subjectively, quite hard to find the working configuration and also it took long, because creating the not working cluster then, resulted in timeout after about 43 min. However, this problem was solved.

### 5.5. Meeting the Automation Requirement

It was extremely important for us to automate the cluster operations. This was needed for several reasons: we wanted to make the experiment easy to be repeated, we wanted to be able to quickly recreate a cluster, and also we wanted to achieve such level of operations automation, so that a CICD server could be used to continuously test such a cluster.

Apart from that, we also decided to follow Infrastructure as Code (IaC) best practices. IaC is an approach which suggests treating everything as code. While kops and eksctl already provide some level of automation, our work resulted in having simple, one-line Bash commands used to (among others) deploy, test and destroy a cluster. For example, in order to deploy an EKS cluster, all what was needed was a git repository https://github.com/xmik/MastersThesis (accessed on 31 January 2021) and the following Bash command:

kops$ ./tasks _create

Then, one had to wait on average 6 min, and the cluster should have been automatically created. The Bash script name was: tasks. Similarly, to test the cluster, one should just run:

kops$ ./tasks _test

The code for the test task can be found here: https://github.com/xmik/MastersThesis/blob/master/src/kops/tasksL165/ (accessed on 31 January 2021) and all the tests are kept here: https://github.com/xmik/MastersThesis/blob/master/src/tests/tests.bats (accessed on 31 January 2021).

We tested for example: whether a correct version of kubectl was installed, whether the worker nodes have status: Ready or whether a test application (a microservice) can be deployed on top of such a cluster. The Bats-core framework was used.

It is worth summarizing how automating the cluster operations progressed. Firstly, both kops and eksctl automatically edited the local kubeconfig files. This was tremendously helpful, because immediately after cluster creation, one can access a cluster using kubectl command.

Secondly, there was a difference concerning the moment in which the cluster creation commands return. The kops creation command returned immediately. This means that in order to automate the cluster creation to the extent that it can be run on a CI server, some waiting mechanism was needed. However, the waiting mechanism was easy to implement, because kops provides the kops validate cluster command. The command was run in a loop (with sleep in between each trial) and after is succeeded, the cluster was deemed created. On the other hand, creating the cluster with eksctl returned after all the AWS resources were created.

Besides the aforementioned differences, automating the operations was done in a very similar manner for both deployment methods. Creating the configuration was harder with kops, but this was a manual step and it does not need to be run on a CI server.

### 5.6. Cost of Chosen Kubernetes Cluster Operations

Thanks to really deploying the clusters, it was possible to be sure of exactly which and how many of AWS resources (such as EC2, ALB, EBS) were created by a deployment tool. Based on this information it was possible to compare how much a Kubernetes cluster deployed with eksctl and kops would cost to run for one month. This information comes from a real-life scenario and could be useful to someone who wants to have a Kubernetes cluster deployed for a longer time.

**Figure 5:**
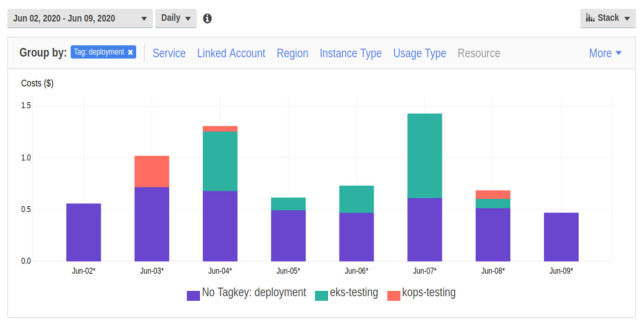
Cost report available on AWS Cost Explorer, grouped by the AWS tag: deployment.

To be aware of the cost generated by AWS resources needed to run the clusters, an AWS tag was applied to all the AWS resources. Thanks to such a solution, it was possible to use the AWS Cost Explorer to verify AWS resources cost. The particular tag applied was either deployment: eks-testing or deployment: kops-testing, depending on the deployment method used. In practice, no cluster was deployed with the label deployment: kops-production or deployment: eks-production, because in this work there was no difference between the testing and production environment. The intention of having two environments was to prove that all the configuration and operations can be automated for multiple environments and, so that in the future, it is possible to choose the environment. Chart taken from the AWS Cost Explorer website is presented in Figure 5.

This chart should not be treated as a single source of truth, because firstly, the AWS tags were enabled later in the empirical work phase and secondly, experimenting with the clusters created with kops took different amount of time and was performed different amount of times than experimenting with the clusters created with eksctl. The chart is just a way of showing an example how it is possible to monitor Kubernetes clusters cost.

To be able to compare the cost of a cluster created with kops and a cluster created with eksctl, the following steps were taken:

1. A cluster was created with kops, using the *src/kops/cluster.yaml* file and central logging was deployed.
2. All the AWS resources decorated with the tag: deployment kops-testing were listed with the following command: *aws resourcegroupstaggingapi get-resources—tag- filters*
  Key=deployment,
  *Values=kops-testing*.
3. The kops cluster was deleted.
4. A cluster was created with eksctl, using the *src/eks/cluster.yaml* file.
5. All the AWS resources decorated with the tag: *deployment eks-testing* were listed with the following command: *aws resourcegroupstaggingapi get-resources –tag- filters*
  Key=deployment,
  *Values=eks-testing*.
6. The AWS EKS cluster was deleted.

This procedure resulted in two lists of all AWS resources, used by each Kubernetes deployment method. Next, all the free AWS resources (VPC Subnets, Security Groups, Internet Gateway, Route Table) were omitted. Overall, using eksctl, the following AWS resources were used: 1 NAT Gateway, 2 CloudFormation stacks, AWS EKS cluster, 2 EC2 instances of type: t2.small (used asworker nodes), an S3 bucket, 1 Load Balancer (ALB) (to expose API server). For kops, there was a bug and not all the AWS resources were tagged [32,42,67]. That bug should be fixed in the future releases of kops, because there is already a PR merged on kops Github.com website [68]. Despite this, it was possible to itemize the not free AWS resources used by kops: 3 EC2 instances of type: t2.micro (used as master nodes), 2 EC2 instances of type: t2.small (used asworker nodes), 1 Load Balancer (ALB) (to expose API server), 6 EBS volumes (3 for etcd-main and 3 for etcd-events) of type gp2 and 20GB each, an S3 bucket. Whenever Elastic IPs were used, they were omitted, because an attached Elastic IP does not cost anything.

Then, the cost for running a cluster for one month was computed. It is presented in Table 3 and Table 4. Each of them lists the AWS resources used by one deployment method. The last row of each table contains the total amount, in USD, of the monthly cost of the AWS resources. The calculated cost does not include everything (e.g., transfer costs), but it should be enough to compare kops to eksctl. Many pricing information is given by Amazon per hour. 720 h in a month was assumed. When it comes to CloudFormation resources, there were two stacks used.

The first stack managed 10 resources and the second stack—34. The CloudFormation pricing [71] depends on operations count. 10 + 34 = 44 creation operations and 10 + 34 = 44 deletion operations were assumed based on the information given. 88 operations total. The pricing website also informs that for operation durations above 30 s per operation, one will be charged $0.00008 per second above the threshold. It cannot be assumed that all the resources took the same time to create, this was not measured. Thus, this cost is not included in the below calculations.

When it comes to the S3 cost, the storage cost is very cheap—$0.023 for 1 GB [73]. The storage used by either kops or eksctl was for sure less than 1 GB, thus $0.023 was assumed as monthly usage cost. Transfer costs were omitted.

Based on the calculations provided above it costs 162.83 dollars to run a Kubernetes cluster created by eksctl and 96.60 dollars to run a Kubernetes cluster created by kops, on AWS, for a 30-day month. Some costs, e.g., data transfer, were not included.

## 6. Discussion and Conclusions

The paper presented the comparison of two methods for Kubernetes cluster deployment, using kops and using eksctl. Seven comparison criteria were applied. The criteria entailed: whether all the production environment requirements were met, time needed to operate a cluster, number of additional prerequisites, minimal EC2 instance types for worker nodes, easiness of configuration, meeting the automation requirement and cost of chosen Kubernetes cluster operations.

Some criteria are subjective, because one may enjoy having a wide choice of possible configuration to set, whereas another (or other use case) would prefer to create a cluster without a redundant hassle. Unquestionably: time and cost are the criteria helping us to decide objectively which method is better. Considering the two criteria, the winner is kops. Kops also provides more configuration options and can be applied on multiple clouds (not only AWS).

On the contrary, eksctl provides an easier way to configure the cluster, because it is convenient to create a YAML configuration file by hand. There are also no prerequisites needed before performing the deployment with eksctl, unless one needs to satisfy the backup requirement. Then, some storage location (such as an S3 bucket) is needed. Kops needs an S3 bucket to keep the cluster configuration. Besides, eksctl provides a managed service (AWS EKS), thus the Kubernetes cluster administrator does not have to worry about managing the control plane components. The last things that should be noted is that eksctl is not the same as AWS EKS, even though eksctl utilizes AWS EKS resource. Furthermore, all the results collected in this study may depend on the versions of kops, eksctl and maybe Kubernetes itself. Our works used the particular versions of each tool.

Concluding each method can be used for different use cases. For example: kops allows more configuration, whereas eksctl is easier to configure. The method which was deemed faster (in relation to cluster operations) and cheaper was kops.

The Kubernetes clusters were deployed in a formal, documented way. First, we gathered the requirements needed for a production environment. We wanted to work on a cluster which is closer to a real-life scenario and represents a more practical approach. Afterwards, the requirements were planned, implemented and the clusters were deployed, tested and destroyed. Our article is based on our deployed clusters, not just on technical documentation.

The comparison of the two deployment methods could hopefully become useful to anyone responsible for a Kubernetes cluster deployment or for planning it. Such a person may learn how much time is needed to deploy a running, production-grade cluster or how easy it is to configure a cluster or how much running such a cluster for one month would cost. This information may influence the deployment decisions and may result in realising a need for more experimentation.

Similarly, the operations automation approach may also be useful for a pragmatic Infrastructure Engineer who wants to limit human errors while deploying a cluster and to ensure a repeatable and easily reproducible process. Our one-line operations are easy to invoke and ready to be used with a CICD server. Based on such a solution, one could expand it by adding new CICD pipeline stages, for example: to test if the cluster can be updated automatically.

Probably the most obvious way to make our comparison better is to add more deployment methods. Kubernetes can be deployed in the cloud, thus other managed services, such as Azure Kubernetes Service (AKS) or Google Kubernetes Engine (GKE) could be tried. Also it could be possible to determine more production environment requirements then nine considered. Ideas for additional requirements are: cluster live upgrades, cluster live reconfiguration, dealing with persistent data, obeying some country laws (concerning for example how long some data should be kept) and more. Moreover, every administrator or every company has their own views on how to satisfy each of the requirements. For some people white listing one IP address to have access to a Kubernetes API server is enough, while the other could demand to keep all master and worker nodes in a private network and use a bastion host. Also the comparison criteria could be performance oriented. In such a case, a chosen test application could have had needs for special resources, maybe running some Machine Learning tasks, demanding access to GPU, lighting fast data transfer and tiny latencies.

By using Kubernetes technology, users can build data science and artificial intelligence platforms based on the best specialized components. With projects such as Kubeflow and Open Data Hub, Kubernetes technology is becoming the standard and primary AI platform.

The scope of artificial intelligence applications in the areas of IT infrastructure operation and Dev (Sec) Ops is expanding, therefore it should be expected that the interest in such solutions will increase in the case of new enterprises with large market opportunities. The core element of such environments will be Kubernetes technology, as it offers the right possibilities for standardization and automation, especially in combination with the concept of Kubernetes operators. Customers can count on an increase in reliability, quality and scalability both in the production environment and in the processes of software development and operation.

Amazon EC2 P4 instances powered by Intel Cascade Lake processors contain NVIDIA A100 Tensor Core GPUs, each connected to the others via NVLink and support NVIDIA GPUDirect. They can deliver up to 2.5 times the performance of deep learning compared to P3 instances. EC2 UltraClusters can withstand the toughest loads in the field of machine learning and HPC on a supercomputer scale: natural language processing, image detection and classification, scene understanding, seismic analysis, weather forecasting, financial modelling.

## Figures and Tables

**Table 1:** Time needed to run Kubernetes management operations using kops and eksctl.

Operation	Using Kops Method	Using Eksctl Method
Create a minimal cluster	6 min 12 s	19 min 26 s
Create a production-grade cluster	6 min 35 s	25 min 40 s
Test a cluster	6 min 33 s	6 min 40 s
Delete a cluster	2 min 23 s	13 min 25 s

**Table 2:** Files used to deploy minimal and full clusters using: kops and eksctl.

Which Cluster	Using Kops Method	Using Eksctl Method
A minimal cluster	src/kops/cluster-minimal.yaml	src/eks/cluster-minimal.yaml
A production cluster	src/kops/cluster.yaml and central logging deployed	src/eks/cluster-minimal.yaml

**Table 3:** Expected cost of running a Kubernetes cluster created with eksctl for one month.

AWS Resource	Cost Using Eksctl Method
1 NAT Gateway [69]	720 × $0.048 = $34.56
Classic Load Balancer [70]	720 × $0.028 = $20.16
2 *CloudFormation* stacks [71]	88 × $0.0009 = $0.0792
AWS EKS cluster [46]	720 × $0.10 = $72
2 *EC2* instances of type: *t2.small* [72]	2 × 720 × $0.025 = $36
keeping files on S3 bucket [73]	$0.023
Total	$162.8222

**Table 4:** Expected cost of running a Kubernetes cluster created with kops for one month.

AWS Resource	Cost Using Kops Method
3 EC2 instances of type: *t2.micro* [72]	3 × 720 × $0.0126 = $27.216
2 EC2 instances of type: *t2.small* [72]	2 × 720 × $0.025 = $36
Classic Load Balancer [70]	720 × $0.028 = $20.16
6 EBS volumes of type gp2 and 20GB each [74]	6 × 20 × $0.11 = $13.2
keeping files on *S3 bucket* [73]	$0.023
Total	$96.599
